# Improving national alignment to mark 50 years of WHO’s essential medicines lists

**DOI:** 10.2471/BLT.26.295706

**Published:** 2026-05-01

**Authors:** Lorenzo Moja, Bernadette Cappello, Deusdedit Mubangizi

**Affiliations:** a Medicines and Health Products Policies and Standards Department, World Health Organization, Avenue Appia 20, 1211 Geneva, Switzerland.

In 1977, the World Health Organization (WHO) published the first *WHO Model list of essential medicines* to determine which medicines were so vital to public health that every health system should ensure their availability.^1^ Developed in response to growing awareness that medicine policies must reflect population needs rather than market forces, the model list aligned with principles later enshrined in the Alma-Ata Declaration.^2^ The model list’s purpose was pragmatic: to support rational selection, efficient procurement and equitable access in settings where resources were, and remain, constrained.

By defining a core set of medicines with proven clinical value, comparative effectiveness, and acceptable safety, the WHO model list created a shared language for clinicians, payers and manufacturers. The model list also offered a template for countries to develop national essential medicines lists tailored to their epidemiological needs and budgetary realities. The early focus on primary care and affordable treatments reflected both the disease burden of the time and the need to build a foundation for universal access.

Fifty years on, the concept of essential medicine remains relevant. The WHO model list has evolved to include new therapeutic classes, biologics and medicines for noncommunicable and rare diseases, while maintaining rigorous standards for evidence and relevance. Initiatives such as the *Lancet* commission on accelerating progress on essential medicines explore how the WHO model list can continue to guide countries towards universal health coverage, emphasizing prioritization of medicines at country level based on clinical value and public health impact across all income levels.^3^ WHO collaborating centres are also preparing for the model list’s fiftieth anniversary with regional and editorial initiatives.^4^

Nonetheless, governments must be held accountable for ensuring their national lists are up to date and aligned with the principles of the WHO model list. Although Member States formally endorsed the essential medicines concept at the 1978 World Health Assembly, national adoption remains uneven.^5^

Some variation between national lists and the WHO model list is expected, reflecting local epidemiology and health system priorities. However, divergences rooted in outdated procurement habits, ineffective or suboptimal medicine choices, or irregular updates undermine the selection framework’s integrity.^6^ Fragmentation in prioritization weakens purchasing power, complicates forecasting and strains supply chains. Smaller markets struggle to attract multiple suppliers of quality-assured medicines, limiting competition and increasing the risk of shortages.

Moreover, the quality of many national lists is inadequate. Recent studies show that many lists lack details on formulations and treatment indications, impairing their function as procurement tools. National lists should specify recommended alternatives within a therapeutic class and state indications to support volume planning. For example, the WHO model list includes glucagon-like protein-1 receptor agonists specifying which agents (semaglutide, liraglutide, dulaglutide and/or tirzepatide), which formulations and strengths (for semaglutide: injection 0.68 mg/mL, 1.34 mg/mL and 2.68 mg/mL) and for which indications (for example diabetes, in patients with coexisting cardiovascular disease and obesity) they are recommended.^7^ Indications are critical, as they determine the eligible patient population and procurement volume. Extending this class to patients with obesity alone on national lists is legitimate but entails an increase in costs, as it expands the eligible population, leading to higher demand, greater procurement volumes and a rapid rise in overall expenditure.

As the fiftieth anniversary of the WHO model list approaches, requesting greater accountability from national decision-makers is important. Rather than expanding lists indiscriminately or making symbolic additions that may not align with budgetary realities, policy-makers need to reassess how national lists can better serve public health goals. Countries may wish to explore how clearly defined indications can help transform their lists into effective policy tools. Clarifying formulations, such as dosage forms, strengths and delivery systems, could support more efficient procurement and use. Identifying age-appropriate formulations to help stimulate the availability of child-friendly products, would also be valuable.^8^ Making national lists publicly accessible and governed transparently can foster alignment among clinicians, patients and suppliers. Importantly, doing so is a shared endeavour: all countries, regardless of income level, stand to benefit from maintaining a clear, current and functional essential medicines list. Paradoxically, many high-income countries still lack formal, public national lists. WHO encourages countries to consider the fiftieth anniversary of the WHO model list as an opportunity to reflect on increasing total pharmaceutical expenditure and to renew the commitment to medicine policies aimed at stabilizing prices.

The WHO model list remains a living instrument that helps countries focus resources to where they can do the best; the list supports stewardship and appropriate use, and provides a credible anchor for procurement strategies, guideline development and government accountability.
